# ORACLE: Object-Centric Autonomous Coverage Exploration Planner for Discrete Trunk Inspection Under Canopy

**DOI:** 10.3390/s26123785

**Published:** 2026-06-14

**Authors:** Juqi Wei, Hai Wang

**Affiliations:** School of Automotive and Traffic Engineering, Jiangsu University, Zhenjiang 212013, China; wei_ju_qi@163.com

**Keywords:** autonomous exploration, coverage path planning, object-centric planning, UAV inspection, ATSP, SOP

## Abstract

Autonomous inspection of discrete obstacles (e.g., tree trunks in orchards and forests) requires UAVs to visit every target with proper observation distance and heading, while simultaneously exploring the unknown environment. Existing space-guided exploration methods focus on eliminating unknown space and are inherently agnostic to the inspection targets themselves, leading to incomplete coverage and redundant traversal. We observe that the obstacles themselves encode the spatial topology of the environment and can serve as natural planning anchors. Based on this insight, we propose ORACLE, an Object-centric Autonomous Coverage Exploration framework that shifts the planning paradigm from space-guided to target-guided exploration. ORACLE integrates: (1) an online target detection and persistent identification module via occupied-voxel connected component labelling, (2) a density-aware global coverage planner that modulates ATSP costs to prioritize target-dense regions, and (3) a target-guided local planner that replaces frontier viewpoints with direct obstacle observation points in a Sequential Ordering Problem formulation. Experiments in two point-cloud environments reconstructed from real-world forests with contrasting tree densities (Environment I: 50 trunks, $\bar{n}=1.56$; Environment II: 70 trunks, $\bar{n}=2.19$; both with non-uniform spacing) show that ORACLE achieves 98.8% and 99.7% target coverage compared to 22.7% and 25.1% for the space-guided baseline, while reducing the mission overhead ratio from 202.9% to 129.2% (Environment I) and from 176.8% to 126.6% (Environment II). Ablation studies confirm that zone reactivation is the decisive factor for coverage completeness (−18.8 and −17.2 percentage points when disabled in Environments I and II, respectively) and that density weighting improves path efficiency.

## 1. Introduction

Individual-tree-level parameter acquisition in precision forestry and precision agriculture—diameter at breast height (DBH), tree height, stem taper curve, crown structure, among others—underpins forest ecological inventory and agricultural management monitoring [1,2]. Accomplishing these tasks inherently requires the sensing platform to visit each target trunk individually at close range and from appropriate viewing angles, a demand that is especially acute in obstacle-dense, GPS-denied environments such as under-canopy forests and orchards.

At the forefront of forest mensuration, terrestrial laser scanning (TLS) delivers high-quality individual-tree data but is constrained by static viewpoints and low efficiency; mobile mapping systems (MMS) improve efficiency at the cost of data quality [3]. Under-canopy UAV laser scanning (under-canopy ULS) combines the advantages of both and has demonstrated individual-tree measurement accuracy comparable to TLS in boreal forests of Finland. Hyyppä et al. [1] employed a SLAM-integrated under-canopy UAV equipped with a rotating LiDAR sensor for close-range scanning, achieving a 93% trunk detection rate with a DBH estimation RMSE as low as 0.60 cm in sparse pine stands; their follow-up study [2] further validated the feasibility of simultaneously deriving canopy height and stem volume from a single sensor (volume estimation relative standard error ≈ 10%), demonstrating that forest inventory requirements can be met without fusing separate ground TLS and aerial UAV datasets. Liang et al. [3] developed a fully automated under-canopy ULS system and systematically compared the measurement accuracy of ULS with TLS, personal laser scanning (PLS), and other close-range sensing systems. These achievements convincingly demonstrate the value of close-range UAV observation for precision forestry. Nevertheless, all of the above under-canopy flights relied on manual remote piloting [1,2] or possessed only basic autonomous traversal capability [3], making them time-consuming, labour-intensive, and limited in spatial coverage. On the autonomy front, Jarin-Lipschitz et al. [4] achieved real-time replanning at 2.5 m/s in real pine forests through a search-based planning framework with adaptive sampling density, demonstrating the feasibility of fast autonomous flight in dense woodland—yet their focus was on point-to-point safe navigation rather than target-oriented coverage planning. Jelavic et al. [5] integrated mapping, localization, planning, and control into a fully autonomous system operating in a GPS-denied real forest, completing selective tree-by-tree harvesting with a full-size hydraulic legged harvester through a pipeline spanning mapping, tree selection, autonomous navigation, and grasping—demonstrating the engineering viability of precision single-tree operations, albeit with pre-built maps and manual tree selection rather than online discovery. As Liang et al. explicitly stated, “autonomous path planning in complex unknown under-canopy environments remains a critical bottleneck for practical deployment of such systems” [3].

Meanwhile, orchard inspection and management in precision agriculture face analogous challenges. Ground robots must navigate autonomously between fruit tree rows and perform spraying, monitoring, and other operations on each individual tree [6,7], while aerial UAVs must weave among trees to accomplish coverage monitoring [8]. Chatziparaschis et al. [6] achieved on-the-go real-time tree detection and geometric parameter estimation (width, height) on a ground mobile robot through multi-modal fusion of NDVI imagery and LiDAR point clouds. Jiang et al. [9] detected tree trunks from 2-D LiDAR point clouds using DBSCAN, K-means, and RANSAC and realized inter-row navigation (detection success rate > 95%, lateral RMSE ≈ 12 cm). Li et al. [10] proposed an octree-based adaptive 3-D point cloud compression method that reduces data volume by approximately 76% while preserving critical obstacle information, thereby enabling real-time RRT* planning. Xu et al. [7] pre-detected fruit tree positions from UAV imagery and planned global navigation paths, following a “perceive first, plan later” offline paradigm. These works contribute rich domain knowledge and engineering validation, yet they either assume a known environment (offline planning) or rely on the prior assumption of regular planting rows for inter-row navigation, limiting their adaptability to unstructured woodland; none address the core problem of “online discovery and efficient traversal of discrete targets in unknown environments”.

Taken together, these studies point to a critical need: whether in forest mensuration or orchard management, multi-rotor UAVs or ground robots must autonomously navigate beneath the canopy without prior maps, visiting every discrete trunk target at appropriate observation distances and viewing angles, while performing online incremental target discovery, tracking, and real-time observation path replanning during exploration. The primary evaluation metric for such tasks is not “how much unknown space has been explored” but rather “how many target trees have been effectively visited”. When prior maps are unavailable, fully autonomous coverage exploration that couples online target discovery with traversal planning remains an open research problem.

Over nearly three decades of development, autonomous exploration has converged on a mature space-guided paradigm. From Yamauchi’s seminal introduction of the frontier concept (the free–unknown boundary) that established the classical framework for frontier-based exploration [11], through information-gain-driven methods and the hierarchical planning frameworks exemplified by FUEL [12], TARE [13], and GBP [14] to the recent coverage-path-guided frameworks represented by FALCON [15] and FLARE [16], spatial exploration efficiency has steadily improved (detailed analysis of individual methods is provided in Section 2.1). However, these methods have invariably focused on how to eliminate unknown voxels as quickly as possible; their shared implicit assumption is that “eliminating unknown voxels equals task completion” rather than “maximizing target coverage”.

When the task objective shifts from spatial exploration to discrete obstacle inspection, this assumption reveals two structural deficiencies: (1)  Target omission—Once the unknown space in a region has been eliminated, the space-guided planner marks the region as “completed” and redirects the UAV, even if inspection targets within that region have not been visited at appropriate distances and angles, causing targets to be systematically overlooked. (2)  Path guidance bias—Frontier viewpoint selection optimizes only in the direction of information gain, greedily chasing locations that reveal the most unknown space without considering the spatial distribution of actual targets; this misalignment between the global guidance direction and the positions of targets that need to be visited produces substantial redundant backtracking trajectories. In short, space-guided methods do not know “what in the environment is worth visiting”—they only know “where unknown space remains to be explored”.

We observe that, for such discrete obstacle inspection tasks, the obstacles themselves encode the topological structure of the operational space—the spatial distribution of tree trunks defines navigable corridors, regional target densities, and reasonable traversal orders. This structural information is entirely disregarded under the space-guided paradigm: the voxel map merely distinguishes free, occupied, and unknown states, without knowing “which occupied voxels belong to the same tree,” let alone “whether that tree has already been effectively visited”. Shifting the planning focus from “viewpoints targeting unknown space” to “obstacle targets” therefore addresses both structural deficiencies simultaneously: the planner directly pursues the actual inspection objective and inherits the environmental structure encoded in the obstacle distribution, aligning global guidance with local execution.

Building on this insight, we propose an **O**bject-cent **R**ic **A**utonomous **C**overage exp **L**oration fram **E**work (ORACLE), an autonomous coverage exploration framework centred on the objects to be visited, shifting the planning paradigm from space-guided to target-guided exploration. ORACLE performs a fundamental paradigm extension upon the hierarchical exploration framework of FALCON [15]. FALCON partitions the environment into functional zones through incremental connectivity-aware space decomposition and generates a global coverage path via inter-zone ATSP, which then constrains a SOP for local viewpoint traversal, pursuing spatial coverage efficiency. ORACLE retains this hierarchical architecture but redirects its objective from spatial coverage to target traversal—transforming “using ATSP/SOP to accelerate spatial exploration” into “using ATSP/SOP to achieve target traversal coverage”—by injecting target-guided capabilities at three key levels: online target detection and persistent identity management at the perception layer, density-aware zone reactivation and cost modulation at the global planning layer, and observation-point-based target traversal at the local planning layer (detailed in Contributions C1–C3 below). This design enables ORACLE to acquire the capability for discrete target coverage traversal while preserving efficient spatial exploration, yielding a unified coverage framework that simultaneously addresses spatial exploration and target traversal.

The main contributions of this paper are as follows:

1. An object-centric autonomous coverage exploration paradigm. We propose the ORACLE framework, which shifts the planning objective of autonomous exploration from eliminating unknown space to traversing discrete obstacle targets, achieving a paradigm shift from space-guided to target-guided exploration upon FALCON’s coverage-path-guided architecture.
2. Online target detection and incremental identity management via occupied-voxel CCL. Exploiting the geometric prior of tree trunks (approximately vertical cylinders), we perform online trunk detection through z-slab projection, eight-connected BFS flood fill, and a three-stage filtering pipeline, combined with greedy nearest-neighbour matching and global overlap merging for cross-frame persistent identification, global deduplication, and real-time visit status determination, providing a stable global target list for subsequent target-guided planning.
3. Density-aware hierarchical coverage path planning. At the global (ATSP) level, target-driven zone reactivation and a β-modulated density cost weighting function steer the path to preferentially traverse target-dense regions; at the local (SOP) level, trunk observation points replace frontier viewpoints and the same density weighting is applied, achieving semantically consistent target-guided planning across both hierarchical levels.
4. Experimental validation in real forest point cloud environments. In simulated environments reconstructed from real forest point clouds with varying tree density distributions, ORACLE significantly outperforms space-guided baselines in both target coverage rate and path efficiency; ablation studies verify the independent contribution of each module.

## 2. Related Work

ORACLE seeks to perform simultaneous spatial exploration and discrete-target traversal in a completely unknown environment—an objective that cuts across several research communities. This section reviews three interrelated technical threads: we first trace the evolution of UAV autonomous exploration (Section 2.1), showing how space-guided exploration has advanced from greedy strategies to coverage-path-guided hierarchical systems, yet has never incorporated physical inspection targets into the planning loop; we then examine the theory and methods of coverage path planning (CPP) (Section 2.2), noting that coverage objects remain geometric areas or grid cells without coupling to online target discovery; and we finally discuss target traversal planning and semantic-aware exploration (Section 2.3), analysing the gaps between existing methods—which assume known target locations, optimise for reconstruction quality, or rely on heavyweight detection pipelines—and the per-target traversal completeness required for under-canopy inspection. Together, these three threads converge on a problem that has not been systematically addressed: how to discover discrete obstacle targets online during exploration and seamlessly integrate them into a combinatorial optimisation framework for efficient and complete traversal.

### 2.1. Autonomous Exploration

Since Yamauchi introduced the frontier method [11], UAV autonomous exploration has undergone roughly three generations of evolution. The first generation of greedy frontier methods selects the nearest or highest-information-gain frontier to visit one at a time, resulting in myopic decisions and frequent long-distance backtracking. The second generation of information-gain and hierarchical planning methods sought to extend the evaluation horizon to multi-step sampling: Dai et al. [17] proposed a frontier–sampling hybrid strategy with implicit octree-based grouping, achieving fast information-driven MAV exploration; Selin et al. [18] was among the early works to combine global frontier exploration planning (FEP) with a local receding-horizon next-best-view planner (RH-NBVP), achieving complementary inter-region navigation and intra-region fine-grained exploration; FUEL [12] introduced the incremental frontier information structure (FIS) to cluster frontiers into frontier groups, coupled with a three-tier “global coverage–local path–minimum-time trajectory” planning pipeline, improving exploration efficiency by 3–8× over prior methods; GBP [14] reused graph structures for global exploration path search and validated air–ground heterogeneous platform cooperative exploration in the DARPA SubT underground challenge; and TARE [13] proposed a “local dense/global sparse” dual-layer representation strategy that reduces computational cost by over 50% and improves coverage efficiency by 80% in complex 3-D environments.

Building upon this foundation, the third generation of coverage-path-guided methods introduced combinatorial optimisation into exploration frameworks to enhance global path efficiency. FAEP [19] formulated frontier groups as TSP nodes to solve the global visitation order and designed a two-stage heading planner to maximise en-route frontier observation coverage during each flight. FALCON [15] proposed incremental connectivity-aware space decomposition to partition the environment into functional zones and construct a connectivity graph, using an inter-zone ATSP coverage path as global guidance that constrains a SOP for local viewpoint traversal—reducing exploration time by 13.8–29.7% compared to the best baseline across six benchmark scenarios. FLARE [16] shifted the planning focus from frontier boundaries to “unknown regions,” treating unexplored space (rather than merely frontier boundaries) as the global planning object, incrementally segmenting unexplored space into independent regions with explicit partition-based guidance to improve global path quality (exploration time reduced by 16.8–27.9%, flight distance reduced by 15.8–25.5%). Ye et al. [20] introduced boundary-aware heuristics and adaptive sampling into a TSP + LKH two-stage framework for real-time spatial mapping. EDEN [21] proposed a dual-layer planning architecture with curvature-penalty scoring to reduce sharp-turn deceleration, enabling high-speed regional routing in large-scale environments.

These methods continue to push the upper bound of spatial exploration efficiency. However, their TSP/ATSP nodes remain centroids of spatial regions or representatives of frontier viewpoint clusters—encoding “where unknown space exists” rather than “where inspection targets are located”. Their core optimisation objective is therefore always to eliminate unknown voxels as quickly as possible, without regard for specific physical targets in the environment, and they offer no coverage completeness guarantee for physical targets. When the task shifts from spatial coverage to discrete target traversal, these methods lack the ability to incorporate online-discovered targets into their combinatorial optimisation frameworks.

### 2.2. Coverage Path Planning

Coverage path planning (CPP) aims to generate a path that enables a robot to traverse a designated area or target set. Jayalakshmi et al. [22] systematically surveyed recent advances in CPP in a review spanning 237 publications, categorising existing methods into geometric decomposition, grid decomposition, bio-inspired, and learning-based approaches, among others. The classical Boustrophedon (lawnmower-style) decomposition segments a known environment into convex sub-regions and executes back-and-forth sweeps within each; Bähnemann et al. [23] reformulated Boustrophedon coverage as a generalised TSP (GTSP), optimising the inter-subregion visitation order with a GTSP solver and reducing path cost by 14%.

In the area of constrained coverage, Jensen-Nau et al. [24] proposed the Voronoi-based path generation (VPG) algorithm, modelling paths as a mass–spring–damper network to achieve near-optimal area coverage under energy constraints while maintaining linear time complexity. Schweppe et al. [25] further formulated coverage planning as an MPC online optimisation problem, allowing different regions to carry different coverage weights such that high-weight regions are covered preferentially—a “weighted coverage” concept that resonates with ORACLE’s target-density-aware cost modulation in motivation.

In the context of online coverage in unknown environments, Kan et al. [26] proposed hierarchical hexagonal decomposition for online coverage planning that requires no environmental prior and performs coverage concurrently with exploration, an early representative of the “explore-while-covering” strategy, though its coverage object remains area rather than discrete entities. Bouman et al. [27] leveraged submodularity modelling to achieve adaptive sequential coverage–exploration decision-making in the DARPA SubT competition. Li et al. [28] designed a multi-UAV target coverage path planner for dynamic environments using greedy assignment and ACO with variable pheromone.

In the domain of multi-region coverage, Xie et al. [29] were the first to formalise the coupled TSP–CPP problem—jointly modelling and optimising the global visitation order across multiple discrete regions (TSP) together with the intra-region coverage path (CPP)—a hierarchical “global ordering + local coverage” concept that serves as a direct theoretical precursor to ORACLE’s hierarchical planning architecture (global ATSP for region visitation order, local SOP for target traversal).

The CPP methods reviewed above provide a rich body of coverage optimisation theory and engineering precedents, yet they share a common limitation: most assume a known environment (offline planning) or adopt geometric regions or grids as the coverage objective, without linking to specific physical inspection targets. Even those that begin to handle dynamic information or multi-region joint optimisation do not address the coupled problem of “online discovery of discrete obstacle targets in a completely unknown environment and their individual coverage traversal”.

### 2.3. Target Visiting Planning and Semantic-Aware Exploration

When target locations are known, multi-target traversal can be directly modelled as a TSP or its variants and efficiently solved by solvers such as LKH. Helsgaun [30] demonstrated that the LKH solver can produce high-quality solutions within reasonable time even for very large-scale GTSP instances with 17,180 clusters and 85,900 vertices. Guo et al. [31] addressed the multi-target coverage traversal problem by constructing a fast connectivity graph via ISODATA clustering and a connected-component-based mPRM algorithm, then solving a TSP on the connectivity graph to obtain the coverage visitation sequence, substantially improving computational efficiency. In the area of online informative path planning (IPP), Moon et al. [32] proposed IA-TIGRIS, which incrementally reuses historical planning trees and adaptively adjusts the sampling strategy according to an updated belief map for online informative path planning, validating a 38% information gain improvement on hexarotor and fixed-wing UAVs—though its planning targets are provided by an external belief map rather than discovered online.

The common prerequisite of these methods is that target locations are either fully known (TSP traversal) or continuously updated by an external system (belief map in IPP). In scenarios where target locations are unknown and must be discovered in real time during exploration—precisely the situation faced in under-canopy trunk inspection—how to couple target discovery with traversal planning is an open problem that none of these methods addresses.

Some target search works attempt to bridge this gap. Zhang and Yuan [33] proposed an autonomous target search method for limited-FOV camera UAVs that integrates exploration and target search through a visual information gain-aware framework, establishing a logical visit order for frontier viewpoints and generating minimum-time trajectories—yet their planning strategy remains frontier-based, with the global guidance direction driven by spatial information gain rather than target distribution. Wang et al. [34] designed a semantic-aware measurement-driven three-stage autonomous search framework that combines YOLOv8 object detection with DBSCAN clustering for target localisation, employing a semantically-aware dynamic RRT at the local level and TSP-optimised target visit order at the global level. However, the global relocation phase of this method is based on frontier clustering, so the global guidance remains spatially driven and does not provide a complete traversal guarantee for the discrete target set.

Rather than treating targets as add-ons to frontier-based exploration, a more fundamental approach is to embed object-level semantics directly into the exploration decision loop. Semantic-aware exploration is the research direction conceptually closest to ORACLE. Dang et al. pioneered the injection of semantic information about environmental objects into exploration decisions in two 2018 works: one used a visual saliency model to guide receding-horizon planning, directing attention toward “meaningful” objects during exploration [35]; the other introduced dual-gain optimisation (spatial expansion gain + object re-observation resolution gain) to simultaneously perform object search during autonomous exploration [36], demonstrating the value of attending to specific targets during exploration—though both operate within a sampling-tree framework. Papatheodorou et al. [37] further proposed a semantic object-centric MAV exploration framework that balances exploration efficiency and target reconstruction quality in unknown environments—applying a maximum observation distance constraint on background surfaces to prevent target omission, and a smaller observation distance on discovered objects to ensure reconstruction accuracy. This work is the closest to ORACLE in its “object-centric target-guided exploration” planning philosophy and “observation distance constraint” design, but its optimisation objective is to discover objects and reconstruct them at a target accuracy (continuous reconstruction quality optimisation), targeting generic semantic scenes; it does not maintain an explicit target list or visit status, thus providing no guarantee that every target in a specific target set has been effectively visited. Ding et al. [38] proposed the structure-aware topological exploration framework SATE, which utilises U-Net semantic analysis to generate traversability heatmaps, anchors paths to the geometric medial axes of safe regions via semantic-seeded Voronoi decomposition, and introduces topological sparsity regularisation to suppress redundant backtracking, reducing path length by 45% compared to geometric baselines—yet its semantic information is used to guide path topology rather than to identify and traverse discrete targets. Liu et al. [39] demonstrated large-scale autonomous flight and real-time semantic SLAM under dense canopy, detecting tree trunks and ground planes from LiDAR data, associating constraints across scans, and employing a drift compensation mechanism to ensure accuracy over large-scale missions, thus achieving autonomous semantic map construction of user-specified regions. However, their planning still targets spatial coverage (constructing a semantic map of a specified ROI) and does not optimise for complete traversal of discrete targets.

The semantic methods reviewed above corroborate that object-centric planning can improve task relevance, yet three critical differences separate them from ORACLE. First, the optimisation objectives differ: semantic-aware exploration typically optimises reconstruction quality (object surface completeness, semantic map accuracy), a continuous information gain optimisation problem, whereas ORACLE pursues per-target coverage traversal completeness, a combinatorial traversal problem. Second, the target detection approaches differ: most methods rely on deep-learning semantic segmentation models, posing deployment challenges on computation-constrained under-canopy UAV platforms; ORACLE leverages voxelised occupancy information and the geometric prior of tree trunks (approximately vertical cylinders) for purely geometric lightweight detection, avoiding dependence on GPU inference. Third, the coverage completeness guarantee mechanisms differ: semantic exploration methods implicitly “cover” objects through information gain decay but do not guarantee that every target is effectively visited at a prescribed distance and angle; ORACLE provides deterministic coverage completeness guarantees through explicit three-condition visit determination and global target list maintenance.

In summary, the three technical threads reviewed above—autonomous exploration, coverage path planning, and target traversal with semantic exploration—delineate the complete research landscape relevant to ORACLE. Autonomous exploration provides efficient hierarchical frameworks under space-guided principles but lacks target awareness; CPP supplies coverage optimisation theory and online planning tools but takes area or grid cells as coverage objects; target traversal methods assume known targets or external provision, while semantic exploration comes closest conceptually but its optimisation objective favours reconstruction quality over traversal completeness. No existing method systematically unifies online target discovery, global traversal planning, and local coverage execution within a single object-centric framework. ORACLE addresses this fundamental gap with an integrated solution: rather than appending object attention as an auxiliary optimisation to the exploration process, it transforms obstacles from passively discovered entities into the core objects of the planning system—introducing online target detection and persistent identification atop an efficient spatial exploration framework, shifting the planning objective from spatial coverage to target traversal coverage, with target detection, global guidance, and local execution all designed in a unified obstacle-centric manner, realising a unified framework for “online discovery of discrete targets in a completely unknown environment and their efficient complete traversal”.

## 3. System Overview

The preceding sections motivated target-guided exploration at an intuitive level and surveyed related work. This section provides a complete overview of ORACLE from two perspectives: problem definition and system architecture. Section 3.1 establishes a mathematical formulation covering environment representation, target modelling, and the optimisation objective, providing a traceable formal foundation for every module designed in subsequent sections. Section 3.2 presents the three-layer pipeline architecture—perception, global planning, and local planning—together with its event-driven replanning loop, allowing the reader to develop a holistic understanding of the system-wide data flow and inter-module relationships before delving into module-level details.

### 3.1. Problem Definition and Modelling

This subsection translates the problem into a mathematical formulation: we first define the environment and target representation, then state the optimisation objective under an incrementally revealed target set. These definitions directly map to the design of subsequent modules: Section 4 addresses “how to obtain ${\hat{T}}_{t}$ online,” and Section 5 addresses “how to minimise L(π) while guaranteeing full target coverage”.

The UAV operates within a 3-D voxel map constructed in real time from onboard sensors. The environment contains a finite set of discrete obstacles (tree trunks) that are invisible before the mission begins and must be discovered incrementally during exploration. We model each trunk as a cylindrical primitive characterised by its centroid and bounding-circle radius—a simplification that captures the essential geometry of tree trunks while remaining computationally efficient for online detection.

**Definition** **1.**

- *3-D voxelised environment $E\subset R^{3}$; each voxel v has state ∈{free,occupied,unknown}.*
- *Discrete obstacle (tree trunk) set $T=\{T_{1},\ldots ,T_{N}\}$, where $T_{i}=\left(c_{i},r_{i}\right)$ with centre $c_{i}\in R^{3}$ and minimum bounding-circle radius $r_{i}$.*
- *T is unknown a priori; N is likewise unknown to the planner.*
- *The discovered subset ${\hat{T}}_{t}\subseteq T$ grows monotonically during flight.*

Given the environment and target model above, the inspection objective is to minimise the UAV flight path length while ensuring every target is effectively visited (the precise visit conditions are defined in Section 4.3). This formulation treats complete target coverage as the hard task requirement and uses path length as the planner-level efficiency objective; under fixed speed and acceleration limits, reducing traversal length also reduces mission time in practice, without introducing an additional user-defined partial-coverage threshold into the problem statement. However, because the target set T is entirely unknown at the start and can only be revealed incrementally through exploration, the problem cannot be solved by offline global optimisation.

Formally, we seek a flight trajectory π that minimises total path length L(π) subject to full coverage:

(1) $$\min_{\pi }\;L\left(\pi \right)\;s.t.\;\frac{\left|\{T_{i}\in T:T_{i}.\mathrm{status}=\mathrm{VISITED}\}\right|}{\left|T\right|}=100\%$$

Because T is incrementally revealed, a global optimal solution is intractable. ORACLE therefore pursues an online strategy: given the currently discovered targets ${\hat{T}}_{t}$, repeatedly perform region-level global guidance (ATSP) and target-level local execution (SOP) until all targets are visited. In practice, near-complete coverage (e.g., ≥98%) is achieved, with the small residual misses typically attributable to occasional detection failures or identity-matching failures.

### 3.2. Proposed Framework Overview

**Figure 1:**
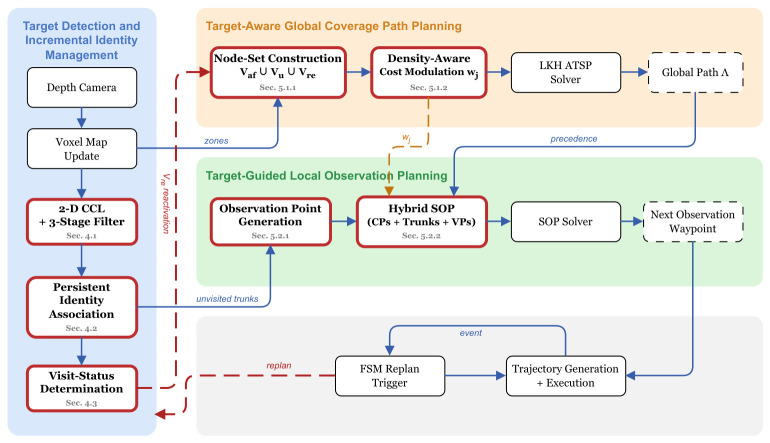
System overview of ORACLE. The three-layer pipeline—perception, global planning, and local planning—is orchestrated by an event-driven FSM. Arrows indicate data flow between modules; section labels refer to the corresponding methodological details.

Figure 1 presents the system architecture of ORACLE, which follows a three-layer pipeline—perception, global planning, and local planning—orchestrated by an event-driven finite state machine (FSM). The perception layer updates the 3-D voxel occupancy map at each planning iteration, then runs 2-D connected component labelling (CCL) on occupied voxels within a designated height slab to extract candidate trunks (Section 4.1), matches them against a persistent global target list via greedy nearest-neighbour association to maintain persistent identity (Section 4.2), and continuously evaluates each target’s visit status during flight (Section 4.3). The global planning layer builds a region-level coverage path on top of this perception output. ORACLE partitions the planning space into uniform grid cells, each of which is further subdivided into zones by spatial connectivity. When a zone has lost all its frontiers (boundaries between known and unknown space)—meaning its space is fully observed—yet still contains unvisited trunks, ORACLE reactivates it into the planning node set. An Asymmetric Travelling Salesman Problem (ATSP) is then solved over these nodes to determine the optimal inter-zone visit order, with zone-based node-set construction (Section 5.1.1) and a density-aware cost weight (Section 5.1.2) steering the solution toward target-dense regions. The local planning layer receives the global ATSP path as an ordering constraint, generates observation points for all unvisited trees in the current and next zone (Section 5.2.1), and formulates them as the primary visit nodes in a Sequential Ordering Problem (SOP); it optionally fuses a small number of en-route frontier viewpoints, then passes the solved hybrid SOP observation sequence to the trajectory generator (Section 5.2.2). During trajectory execution, the system continuously evaluates the three-condition visit criterion for every unvisited target at a sufficiently high frequency; upon satisfaction, the target is marked as visited. When new targets are discovered or local conditions change, the FSM triggers replanning and the three-layer pipeline re-executes. This closed-loop “fly, discover, and replan” mechanism enables ORACLE to achieve ordered traversal of all discrete targets in a completely unknown environment.

## 4. Online Target Detection and Incremental Identity Management

Target-guided exploration presupposes that the system can perceive “where the targets are” and “which targets have already been visited”. However, a standard occupancy grid map only differentiates three voxel states—free, occupied, and unknown—and cannot associate occupied voxels belonging to the same physical object. ORACLE therefore implements an online perception process from raw voxels to planner-ready target entities in three stages: height-slab projection and 2-D connected component labelling aggregate occupied voxels into candidate trunks (Section 4.1), cross-frame greedy matching and global overlap merging assign each candidate a persistent identity (Section 4.2), and continuous three-condition evaluation determines whether each target has been effectively observed (Section 4.3). This process executes at every planning iteration without relying on deep learning or GPU inference—it exploits only the geometric prior that tree trunks are approximately vertical cylinders.

### 4.1. Occupied-Voxel Connected Component Labelling

We exploit a trunk-specific prior: tree trunks form near-vertical cylinders below the canopy. We therefore project occupied voxels in the height slab $z\in [z_{min},z_{max}]$ onto the xy-plane, suppressing canopy and ground clutter. An eight-connected BFS flood fill then segments the projection into connected components $\left\{C_{k}\right\}$, each of which passes through a three-stage filter pipeline: (i) minimum voxel count $|C_{k}|\;\geq n_{min}$, where $n_{min}$ is set to filter fine debris from genuine trunks—at the voxel resolution δ=0.1 m, the smallest expected trunk (r≈0.1 m) projects to a handful of occupied xy-cells, so $n_{min}$ is placed below this count to retain the thinnest detectable stems while rejecting single-voxel noise; (ii) vertical ground-continuity check, which verifies that the component extends downward to the ground plane rather than being a suspended branch or canopy fragment; (iii) bounding-circle radius $r_{k}\in \left[r_{min},r_{max}\right]$, where $r_{min}$ excludes sub-voxel-resolution fragments smaller than any meaningful trunk, and $r_{max}$ excludes multi-trunk clusters and large non-trunk structures while accommodating the largest expected trunk in the target environment. The height slab $z\in [z_{min},z_{max}]$ is positioned between the trunk base and the lower canopy according to the target environment’s morphology, with the lower bound optionally raised to avoid low-lying ground clutter. Surviving components yield target primitives $\left(c_{k},r_{k}\right)$, where $c_{k}$ is the component centroid and $r_{k}$ is the maximum distance from all component voxels to the centroid. Algorithm 1 formalises this procedure.
**Algorithm 1:** Occupied-Voxel 2D CCL with Multi-Stage Filtering**Require:** Occupied voxel set $V_{occ}$ in cell; height slab $\left[z_{min},z_{max}\right]$**Ensure:** 
Target list $\left\{\left(c_{k},r_{k}\right)\right\}$  1:Project: $V_{2D}\leftarrow \left\{\left(v_{x},v_{y}\right)|vs.\in V_{occ},\;v_{z}\in \left[z_{min},z_{max}\right]\right\}$  2:8-connected BFS flood fill → components $\left\{C_{1},\ldots ,C_{M}\right\}$  3:**for** each component $C_{k}$ **do**  4:    **if** $|C_{k}|\;<n_{min}$ **then**  5:        continue {voxel-count filter}  6:    **end if**  7:    Verify ground continuity: check occupied voxels in $\left[z_{gnd,min},z_{gnd,max}\right]$ below $C_{k}$  8:    **if** ground continuity not satisfied **then**  9:        continue {ground-continuity filter}10:    **end if**11:    $c_{k}\leftarrow \frac{1}{|C_{k}|}\sum_{v\in C_{k}}v$ {centroid}12:    $r_{k}\leftarrow \max_{v\in C_{k}}∥v-c_{k}∥+\delta /2$ {bounding radius}13:    **if** $r_{k}\notin \left[r_{min},r_{max}\right]$ **then**14:        {continue radius filter}15:    **end if**16:    Output $\left(c_{k},r_{k}\right)$17:**end for**

### 4.2. Persistent Identity Association

After each cell update, CCL produces a fresh set of components. To maintain one-to-one correspondence with previously detected objects, we associate new components with the existing global list using greedy nearest-neighbour matching with matching threshold $d_{match}=\max\left(5\delta_{res},r_{max}\right)$: matched components inherit the prior status and discovery order; unmatched components receive a new global_id. A post-matching global merge pass fuses any two zones whose centres lie closer than the sum of their radii: the merged centre is the area-weighted (radius-squared) average of the two original centres, the merged radius is their arithmetic mean, and the most permissive visit status is retained (i.e., VISITED if either was VISITED). This guarantees stable, non-duplicated target identities throughout the mission. Algorithm 2 details this procedure.
**Algorithm 2:** Persistent Identity Association**Require:** 
New CCL components $C_{new}$; global target list G; threshold $d_{match}$**Ensure:** 
Updated global target list G  1:// Step 1: Greedy nearest-neighbour matching  2:Sort all $\left(C_{i},G_{j}\right)$ pairs by $∥c_{i}-c_{j}∥$ in ascending order  3:**for** each pair $\left(C_{i},G_{j}\right)$ in sorted order **do**  4:    **if** $C_{i}$ unmatched **and** $G_{j}$ unmatched **and** $∥c_{i}-c_{j}∥<d_{match}$ **then**  5:        Match $C_{i}\to G_{j}$: inherit global_id, status, discovery_order  6:        Update $G_{j}.c$, $G_{j}.r$ from $C_{i}$  7:    **end if**  8:**end for**  9:**for** each unmatched $C_{i}\in C_{new}$ **do**10:    Create new entry in G: assign fresh global_id, status← UNVISITED11:**end for**12:// Step 2: Global overlap merging13:**for** each pair $(G_{i},G_{j})\in G$ where i<j **do**14:    **if** $∥c_{i}-c_{j}∥<r_{i}+r_{j}$ **then**15:        $c_{merge}\leftarrow \left(r_{i}^{2}\cdot c_{i}+r_{j}^{2}\cdot c_{j}\right)/\left(r_{i}^{2}+r_{j}^{2}\right)$16:        $r_{merge}\leftarrow \left(r_{i}+r_{j}\right)/2$17:        $status_{merge}\leftarrow \max\left(status_{i},status_{j}\right)$ {VISITED > UNVISITED}18:        Replace $G_{i}$ with merged result; remove $G_{j}$19:    **end if**20:**end for**

### 4.3. Real-Time Visit Status Determination

Inspection tasks impose explicit quality requirements on observations: the UAV must not only approach a target physically, but must do so within a suitable distance range and with the correct heading so that the onboard sensor acquires valid data. We formalise this as a three-condition visit criterion. A UAV at position *p* with yaw ψ achieves a valid observation of target $T_{i}$ if and only if all three conditions hold simultaneously:

1. Range: $r_{i}+d_{\mathrm{safe}}\leq ∥p_{xy}-c_{i,xy}∥\leq r_{i}+d_{\mathrm{cov}}$, where $d_{\mathrm{safe}}$ is set according to the UAV half-wheelbase to prevent collision during the observation approach, and $d_{\mathrm{cov}}$ is placed well within the depth camera’s effective sensing range.
2. Heading: $|∠\left(c_{i}-p\right)-\psi |\leq \theta_{\mathrm{fov}}/2$, where $\theta_{\mathrm{fov}}/2$ corresponds to half the camera’s horizontal field of view, ensuring the trunk lies within the sensor frame during observation.
3. $T_{i}$ is currently UNVISITED.

During trajectory execution, the system continuously evaluates this criterion for every unvisited target. When all three conditions are simultaneously satisfied, the target is marked as visited. The coverage rate ${\hat{C}}_{t}=\left|{\hat{T}}_{visited}\right|/N_{gt}$ is updated after each status change. The check runs at a sufficiently high frequency to ensure reliable detection even when the UAV traverses the valid observation annulus at moderate speed. Moreover, visit status directly influences planning behaviour: as long as unvisited targets remain in the global list, the system suppresses termination even after all frontiers have disappeared, maintaining the planning loop—thereby providing a system-level guarantee for coverage completeness.

## 5. Density-Aware Hierarchical Coverage Planning

After target detection and persistent identification have been established, one might naturally consider solving a TSP over all discovered trunks to obtain a global traversal path. This approach is infeasible for three reasons. First, the target set is incrementally revealed—each planning cycle operates on a snapshot ${\hat{T}}_{t}\subseteq T$, and any “optimal” path computed now may be invalidated as soon as new targets appear. Second, while visiting known targets, the system must continue exploring unknown space so that additional targets can still be discovered. Third, online replanning must remain computationally affordable. ORACLE addresses this by decomposing the global problem into two hierarchical levels: a region-level global guide (ATSP, Section 5.1) determines which zone the UAV should head to next, and a target-level local executor (SOP, Section 5.2) uses that guidance as an ordering constraint and determines in what order and via which observation points to visit trunks within the current and adjacent zones. Density information is injected at both levels—reducing the arrival cost for target-dense regions and penalising explored empty regions—so that the path is steered toward high-value areas while remaining computationally tractable.

### 5.1. Density-Aware Global Coverage Path Planning

The global coverage path determines in what order the UAV visits the various regions. To construct this path, ORACLE formulates the problem as an Asymmetric Travelling Salesman Problem (ATSP): each region to be visited serves as a node, inter-node costs reflect estimated flight time, and a solver produces the shortest open-loop tour starting from the current position. In pure spatial exploration, the ATSP node set comprises only two region types—active free zones that still contain frontiers and unknown zones that have yet to be explored. In the target traversal task, however, this node set alone cannot guarantee complete target coverage; furthermore, in environments with non-uniform tree distributions, a cost function based purely on flight time cannot distinguish high-value regions (target-dense) from low-value ones (explored and target-sparse), causing the UAV to waste flight budget on non-productive traversals through sparse areas. This subsection therefore introduces two key modifications to the standard ATSP: target-driven zone reactivation (Section 5.1.1) reintroduces fully explored zones that still contain unvisited targets into the node set, ensuring coverage completeness; and density-aware cost modulation (Section 5.1.2) injects target-density information into the cost matrix to steer the route toward high-value regions. We first present the complete ATSP mathematical formulation and then describe each modification in turn.

#### 5.1.1. ATSP Formulation and Target-Driven Zone Reactivation

Given the current UAV position $p_{c}$ and a set of region nodes, the ATSP objective is to find an open-loop tour starting from $p_{c}$ that passes through every node with minimum total cost.

The ATSP node set comprises four element types:

(2) $$V_{atsp}=\left\{p_{c}\right\}\cup V_{af}\cup V_{u}\cup V_{re}\;$$

where $V_{af}$ contains the viewpoint centres of active free zones (regions that still possess frontiers, with each viewpoint centre defined as the mean position of all frontier viewpoint representatives within the zone), $V_{u}$ contains the geometric centres of unknown zones, and $V_{re}$ contains the planning anchors of reactivated zones. The first two types, $V_{af}$ and $V_{u}$, are inherited from the standard spatial exploration framework—active free zones represent known space with remaining observable unknown voxels, and unknown zones represent completely unexplored space. However, these two node types alone are insufficient for complete target coverage: once a free zone loses all its frontiers, it exits the node set, yet it may still harbour unvisited trees. $V_{re}$ is ORACLE’s key extension to address this gap. Before each ATSP construction, the system scans all non-active free zones within each spatial decomposition cell; if a zone contains at least one unvisited tree, it is reactivated and included in the node set. The planning anchor of a reactivated zone is set to the mean XY position of its unvisited trees. Each tree is assigned to the nearest non-active free zone to prevent duplicate activations.

Each entry $C_{cp}\left(i,j\right)$ of the ATSP cost matrix records the estimated flight time from node *i* to node *j*. Inter-node costs are computed via a hybrid path search: voxel-level A* for short-distance pairs and graph-level A* on an incrementally maintained connectivity graph for long-distance pairs [15]. The resulting path is evaluated using a segment-wise constant-acceleration kinematic model [12]. Specifically, collinear consecutive segments are merged to extract a waypoint sequence $\left\{q_{0},q_{1},\ldots ,q_{K}\right\}$. The baseline flight time is $t_{base}=L/v_{max}$, where *L* is the total path length. At each waypoint *k*, the UAV arrives at speed $v_{max}$ along direction ${\hat{d}}_{k-1}$ and must reorient toward ${\hat{d}}_{k}$. Let $\theta_{k}$ denote the turning angle; the component of the incoming velocity projected onto the new direction is $v_{c}=v_{max}\cos\theta_{k}$. When $v_{c}\geq 0$, the vehicle only needs to compensate for the loss between $v_{max}$ and $|v_{c}|$ along the new direction. When $v_{c}<0$, the incoming motion initially opposes the new direction, so the vehicle must additionally brake and reverse along that direction. The resulting turning-time penalty is:

(3) $$\Delta t_{k}=\frac{(v_{max}-|v_{c}{\left|\right)}^{2}}{2\;v_{max}\;a_{max}}+\left\{\begin{array}{cc} 2|v_{c}|/a_{max} & \mathrm{if}\;v_{c}<0\\ 0 & \mathrm{otherwise} \end{array}\right.$$

The total flight time is the maximum of the position cost and the yaw cost, $t_{flight}=\max\left(t_{base}+\sum_{k}\Delta t_{k},\;t_{yaw}\right)$, because translational motion and yaw rotation execute in parallel. The cost of returning to $p_{c}$ from any node is set to zero, converting the closed-loop TSP into an equivalent open-loop tour originating at $p_{c}$.

**Figure 2:**
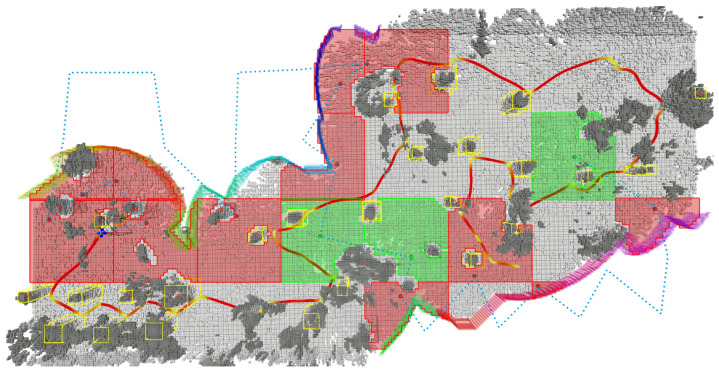
Top-down view of ATSP planning objects. Red-shaded regions denote active free zones; green-shaded regions are fully explored zones where unvisited trees remain (grey bounding boxes)—these are reactivated and injected into the global coverage path (blue dashed lines); grey regions are fully explored and inspected zones (yellow bounding boxes mark visited trees). The actual flight trajectory is colour-coded by speed (red: high speed; yellow-white: low speed). Blank areas traversed by the global path are unknown zones, whose boundaries with known space constitute frontiers.

After density-aware cost modulation (Section 5.1.2), the cost matrix is submitted to the LKH solver [40], which outputs the zone visit order $\bar{\Lambda }=\left\{p_{c},{\bar{z}}_{1},{\bar{z}}_{2},\ldots ,{\bar{z}}_{K}\right\}$. Figure 2 illustrates a representative snapshot of the ATSP planning objects during exploration, showing how reactivated zones are incorporated into the global coverage path.

#### 5.1.2. Density-Aware Cost Modulation

Having ensured completeness of the node set, the next problem is to determine the order in which these nodes should be visited. In environments with non-uniform tree distributions (as is typical of real forests and orchards), a cost function based purely on travel time can lead to repeated traversals through already explored and target-sparse regions—such traversals do not increase the number of visited targets, yet they consume valuable battery and mission time. Intuitively, an efficient strategy should prioritise target-dense regions where a single trip covers many trees, while avoiding regions that are already largely explored and contain no pending targets. ORACLE realises this intuition by applying a column-wise density weight to the ATSP cost matrix. Specifically, every column *j* of the cost matrix (representing arrival costs to destination zone *j*) is multiplied by a weight $w_{j}$. When $w_{j}<1$, the arrival cost is reduced (attraction); when $w_{j}>1$, it is increased (repulsion)—steering the ATSP solution toward allocating more travel to target-dense regions.

Formally, the modulated cost matrix is:

(4) $$C_{cp}^{\prime }\left(i,j\right)=w_{j}\cdot C_{cp}\left(i,j\right),\;\forall i\in V_{atsp}$$

The weight applied to every column *j* is:

(5) $$w_{j}=\frac{1+\beta_{j}}{1+\left(\alpha +\beta_{j}\right)\cdot {\bar{\rho }}_{j}}\;$$

where

(6) $$\beta_{j}=\beta_{0}\cdot \left(1-u_{j}\right)$$

**Figure 3:**
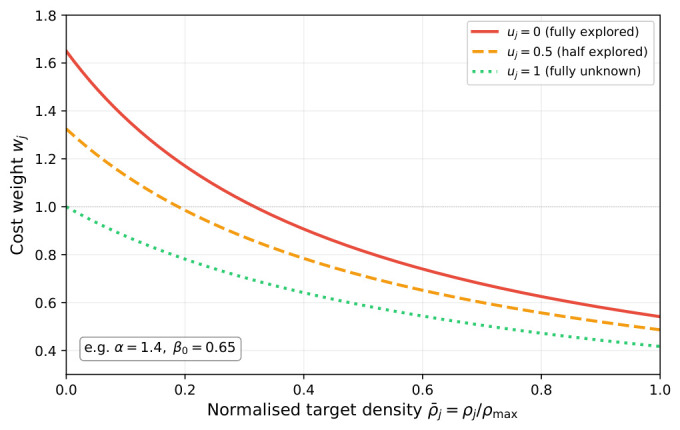
Density-aware cost weight $w_{j}$ as a function of normalised target density ${\bar{\rho }}_{j}$ under different unknown-voxel ratios $u_{j}$.

In the above, ${\bar{\rho }}_{j}$ is the normalised density of unvisited trees in cell *j*, and $u_{j}$ is the fraction of unknown voxels in that cell. The parameter α>0 controls attraction strength: when the target density is high (${\bar{\rho }}_{j}\to 1$), the denominator grows rapidly, pushing $w_{j}$ below 1 and reducing the cost of reaching that region. The parameter $\beta_{0}>0$ controls repulsion strength: when the target density is low (${\bar{\rho }}_{j}\to 0$), $w_{j}$ approaches $1+\beta_{j}>1$, increasing the arrival cost. The term $\beta_{j}=\beta_{0}\cdot \left(1-u_{j}\right)$ introduces a critical exploration-degree modulation—when a cell is largely unexplored ($u_{j}\to 1$), $\beta_{j}\to 0$ and the repulsion effect is disabled, because unexplored regions may harbour undiscovered trunks and should not be prematurely penalised. Only when a region has been thoroughly explored ($u_{j}\to 0$) and confirmed to contain no targets (${\bar{\rho }}_{j}=0$) does the maximum penalty $w_{j}=1+\beta_{0}$ take effect. The same weight formula is also applied to the tree-to-CP block columns of the SOP cost matrix in Section 5.2, achieving semantically consistent density guidance at both the global and local levels. Figure 3 visualises $w_{j}$ as a function of ${\bar{\rho }}_{j}$ under different $u_{j}$ values, showing the attraction–repulsion behaviour and the unknown-ratio modulation.

The values α=1.4 and $\beta_{0}=0.65$ used in our experiments are selected based on a theoretically derived constraint $\beta_{0}<\alpha /2$, which is obtained as follows. A structural property of Equation (5) is that the $\left(1+\beta_{j}\right)$ term in the numerator indiscriminately lifts the weight for all cells with ρ>0, not only empty ones. This creates a “flip point” ${\bar{\rho }}^{*}$ at which $w_{j}=1$, separating attraction ($w_{j}<1$) from unintended penalty ($w_{j}>1$). Solving $w_{j}=1$ yields:

(7) $${\bar{\rho }}^{*}=\frac{\beta_{j}}{\alpha +\beta_{j}}$$

For a cell with the minimum non-zero tree count (ρ=1) to remain in the attractive regime, we require ${\bar{\rho }}^{*}<1/\rho_{\max}$, where $\rho_{\max}$ is the maximum tree count in any single cell. Substituting the worst-case scenario $u_{j}=0$ (fully explored) gives:

(8) $$\frac{\beta_{0}}{\alpha +\beta_{0}}<\frac{1}{\rho_{\max}}\;⟹\;\beta_{0}<\frac{\alpha }{\rho_{\max}-1}$$

In our experiments, $\rho_{\max}=3$ is the most frequent case (accounting for 86% of planning cycles in the representative run log); substituting $\rho_{\max}=3$ yields $\beta_{0}<\alpha /2$. Violating this constraint would mean that low-density cells with one remaining unvisited tree—the most common state in the later stages of a mission—receive a cost penalty rather than attraction, directly contradicting the intended prioritisation of target-dense regions.

With α=1.4, the constraint requires $\beta_{0}<0.7$; we selected $\beta_{0}=0.65$, satisfying the constraint with a small margin. At the critical operating point (${\bar{\rho }}_{j}=1/3$, i.e., ρ=1 with $\rho_{\max}=3$, $u_{j}=0$), $w_{j}\approx 0.98$, a marginal 2% attraction that remains below 1.0 as guaranteed by the constraint. At maximum density (${\bar{\rho }}_{j}=1$, $u_{j}=0$), $w_{j}\approx 0.54$ (46% cost reduction); at zero density with full exploration, $w_{j}=1.65$ (65% penalty); at zero density with complete uncertainty ($u_{j}=1$), $\beta_{j}=0$ and $w_{j}=1.0$. The 46% attraction and 65% repulsion margins are moderate by design: they re-rank planner priorities without overwhelming the baseline flight cost.

A full-scale sensitivity scan over the $\left(\alpha ,\beta_{0}\right)$ space would require hundreds of simulation runs whose volume risks distracting from the paper’s central narrative; likewise, screening combinations to identify a putative optimal region involves intricate multi-objective trade-offs. We therefore adopt a three-part parameter selection strategy: (i) derive a theoretically necessary constraint from the structure of the weight function; (ii) select a parameter pair satisfying this constraint with balanced margins; (iii) verify effectiveness through the controlled ablation study (A2, Section 6.3), where disabling density weighting degrades both efficiency ratios. Systematic parameter sensitivity analysis across diverse environment configurations and the development of automated, possibly online-adaptive, parameter-tuning methods are deferred to future investigation.

### 5.2. Target-Guided Local Observation Planning

The global ATSP determines the inter-zone visit order but does not directly produce an executable observation trajectory—a zone may contain multiple trees requiring close-range observation and several frontier viewpoints worth covering en route, so these targets must be sequenced into a concrete visit order and passed to the trajectory generator. A TSP over the local targets would suffice if all nodes could be visited in arbitrary order, that is, if no precedence constraints existed among them. However, the global ATSP path imposes a zone ordering that the local solution must respect: targets in the current zone should be visited before those in the next zone. This ordering requirement makes the problem a Sequential Ordering Problem (SOP) [41], which extends TSP with precedence constraints. An SOP seeks the minimum-cost permutation of nodes on a directed complete graph G=(V,E) starting from a designated source, subject to a set of precedence constraints P⊆V×V. Formally, let $\sigma =(\sigma_{1},\sigma_{2},\ldots ,\sigma_{\left|V\right|})$ denote a node permutation with $\sigma_{1}$ fixed as the designated source. The SOP objective is:

(9) $$\min_{\sigma }\sum_{k=1}^{|V|-1}C_{sop}\left(\sigma_{k},\sigma_{k+1}\right)\;s.t.\;\forall \left(a,b\right)\in P:\;\mathrm{pos}\left(a\right)<\mathrm{pos}\left(b\right)\;$$

where pos(a) is the index of node *a* in σ. In ORACLE, the precedence constraint set *P* encodes the zone ordering from the global path, ensuring that the local plan does not violate the global guidance.

Unlike the standard spatial exploration framework, where the SOP’s primary visit targets are frontier viewpoint representatives, ORACLE employs tree observation points as the primary planning objects—shifting the local planner’s optimisation objective from “revealing the most unknown space” to “effectively visiting the most tree trunks,” directly aligning with the core inspection requirement. The following subsections describe how observation points are generated (Section 5.2.1) and how the hybrid SOP is assembled with optional en-route viewpoint inclusion (Section 5.2.2).

#### 5.2.1. Observation Point Generation

For each UNVISITED tree $T_{i}$ in the local range (current and next cell), we generate an observation point as follows. The candidate distance is $d_{obs}=r_{i}+d_{offset}$, where $d_{offset}$ is set to place the observation point approximately at the midpoint of the valid observation annulus $\left[r_{i}+d_{\mathrm{safe}},r_{i}+d_{\mathrm{cov}}\right]$, maximising tolerance to trajectory-tracking errors in either direction. Eight candidate directions are defined as $\theta_{k}=\theta_{base}+\Delta_{k}$ where Δ∈{0,±π/4,±π/2,±3π/4,π} and $\theta_{base}$ is the trunk-to-UAV bearing, prioritising an approach from the side facing the UAV so as to reduce detour. The first candidate whose endpoint is a free voxel becomes the observation point $p_{obs,i}$; the associated yaw is $\psi_{obs}=\mathrm{atan}2\left(c_{i}-p_{obs,i}\right)$, pointing directly at the trunk. The *z*-coordinate of $p_{obs,i}$ is set to the current UAV altitude to maintain a consistent flight level. If all eight directions are occupied, the tree is skipped (unreachable). Algorithm 3 formalises this procedure.
**Algorithm 3:** Observation Point Generation**Require:** 
Unvisited trees $\left\{T_{i}\right\}$ in local range; UAV position $p_{uav}$; offset $d_{offset}$; voxel map M**Ensure:** 
Observation point set $O_{tree}$  1:$O_{tree}\leftarrow \emptyset$  2:**for** each unvisited tree $T_{i}=\left(c_{i},r_{i}\right)$ **do**  3:    $d_{obs}\leftarrow r_{i}+d_{offset}$  4:    $\theta_{base}\leftarrow \mathrm{atan}2\left(p_{uav}-c_{i}\right)$ {trunk-to-UAV bearing}  5:    $\Delta_{list}\leftarrow \left[0,\;+\frac{\pi }{4},\;-\frac{\pi }{4},\;+\frac{\pi }{2},\;-\frac{\pi }{2},\;+\frac{3\pi }{4},\;-\frac{3\pi }{4},\;\pi \right]$  6:    found← false  7:    **for** each $\Delta_{k}\in \Delta_{list}$ **do**  8:        $\theta_{k}\leftarrow \theta_{base}+\Delta_{k}$  9:        $p_{k}\leftarrow c_{i}+d_{obs}\cdot {\left[\cos\theta_{k},\;\sin\theta_{k},\;0\right]}^{⊤}$10:        $p_{k}.z\leftarrow p_{uav}.z$ {maintain flight altitude}11:        **if** $M(p_{k})=\mathrm{FREE}$ **then**12:           $\psi_{obs}\leftarrow \mathrm{atan}2\left(c_{i}-p_{k}\right)$ {yaw toward trunk}13:           $O_{tree}\leftarrow O_{tree}\cup \left\{\left(p_{k},\psi_{obs}\right)\right\}$14:           found← true; break15:        **end if**16:    **end for**17:    **if** ¬found **then**18:        Skip $T_{i}$ {all directions occupied; retry at next replan}19:    **end if**20:**end for**

#### 5.2.2. Hybrid SOP Formulation

Replacing frontier viewpoints with tree observation points as the primary SOP nodes introduces a potential side-effect: frontier viewpoints that lie directly along the current flight direction may be silently skipped. If omitted, the UAV may need to backtrack and visit them in a later planning cycle, generating unnecessary return legs. To address this, ORACLE provides a selective en-route inclusion mechanism. When the local range contains at least one unvisited tree and the global path has more than one node, the system screens frontier VP candidates against two conditions: (1) the VP’s Euclidean distance to the UAV is less than the distance to the nearest tree ($d_{vp}<d_{tree,min}$), ensuring that inclusion does not meaningfully extend the path to the nearest trunk; and (2) the VP lies in the forward half-sphere of the UAV’s current heading ($d_{vp}\cdot h_{uav}>0$), ensuring that the selected VP stays aligned with the main flight direction rather than inducing a detour for a single viewpoint. This mechanism preserves the dominance of target-guided planning while incorporating spatial exploration at minimal path cost, reducing the trajectory crossings caused by catch-up visits in later replanning cycles.

Once the observation points and screened frontier viewpoints are ready, they must be assembled together with the coverage-path (CP) node sequence from the global route into a single SOP instance. The SOP vertex set comprises three element types: CP nodes $\Lambda_{cp}$ (*n* nodes) from the global path, tree observation points $O_{tree}$ (*m* points), and screened en-route viewpoints $O_{vp}$ (*v* points), yielding a total dimension of N=n+m+v. The cost matrix $C_{sop}\in R^{N\times N}$ is naturally partitioned into a 3×3 block structure according to the three node types:

(10) $$C_{sop}=\left[\begin{array}{ccc} C^{cc} & C^{ct} & C^{cv}\\ C^{tc} & C^{tt} & C^{tv}\\ C^{vc} & C^{vt} & C^{vv} \end{array}\right]$$

where each superscript pair denotes the source and destination node types (c = coverage-path node, t = tree observation point, vs. = en-route viewpoint). The blocks are defined as follows:

- CP–CP block: $C^{cc}\in R^{n\times n}$: encodes precedence constraints inherited from the global ATSP path. If CP *i* precedes CP *j* in the global route, then $C^{cc}\left(i,j\right)=-1$, instructing the SOP solver to visit *i* before *j*; otherwise $C^{cc}\left(i,j\right)=t_{flight}\left(i,j\right)$.
- Tree–CP block: $C^{tc}\in R^{m\times n}$: each column *j* is multiplied by the density weight $w_{j}$ from Section 5.1.2, i.e., $C^{tc}\left(i,j\right)=w_{j}\cdot t_{flight}\left(i,j\right)$, ensuring that density guidance remains semantically consistent across the global and local levels.
- All other blocks: $C^{\left(\cdot \right)}\left(i,j\right)=t_{flight}\left(i,j\right)$, the estimated flight time between the corresponding node pair.

Before submission to the SOP solver, the row and column corresponding to the “next CP position” in the global path are removed, since that position is an intermediate waypoint that the UAV is about to reach, rather than an independent node to be traversed. The node sequence output by the SOP solver defines the observation visit order within the current local range; its first non-CP node is selected as the next trajectory goal.

## 6. Experiments

To systematically validate ORACLE’s design decisions, the experiments are organised around two progressive questions: first, whether the target-guided paradigm yields measurable coverage and efficiency advantages over the space-guided paradigm (Section 6.2); and second, how much each module contributes independently and how far the actual online path deviates from the offline optimum (Section 6.3, ablation study with path efficiency analysis). Because mission completion requires a precise termination criterion and quantifiable evaluation metrics, we introduce the exploration termination condition and the evaluation metrics as part of the experimental setup (Section 6.1).

### 6.1. Experimental Setup

**Figure 4:**
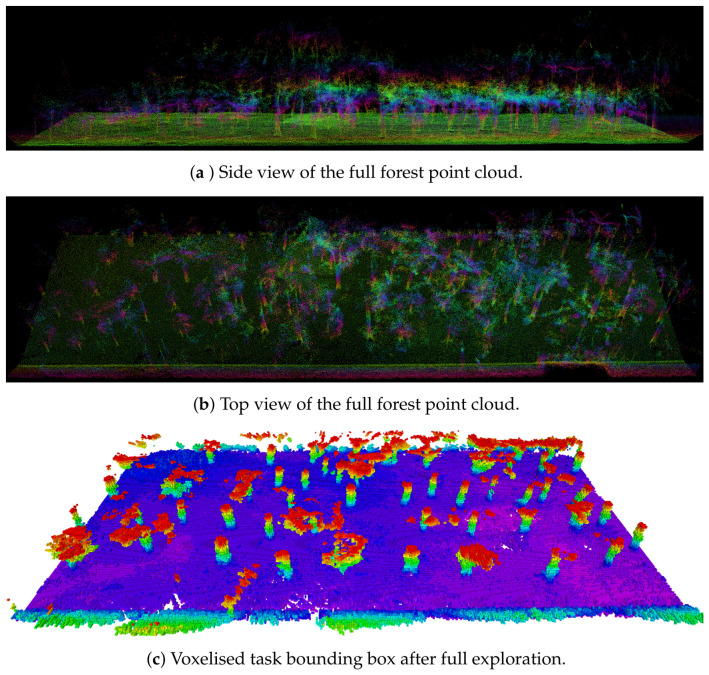
Point-cloud environment I reconstructed from a real forest scene [42]. (**a**,**b**) The full point-cloud map (78m×24m×15.5m) viewed from the side and top, respectively, showing the canopy structure and trunk distribution. (**c**) The voxelised occupancy map of the task bounding box (40m×19m×2m, below canopy) after complete exploration, containing N=50 tree trunks with non-uniform spacing; occupied voxels are colour-coded by height.

All experiments run on an Intel NUC12 mini-PC (Intel Corporation, Santa Clara, CA, USA; Intel Core i5-1240P, 2.1/4.4 GHz, 16 GB RAM) under Ubuntu 20.04 LTS (Canonical Ltd., London, UK) with ROS Noetic (Open Robotics, Mountain View, CA, USA). Environment I is a point-cloud dataset reconstructed from a real-world forest published by MARSIM [42]; the planning bounding box spans 40m×19m×2m and contains N=50 tree trunks with non-uniform spacing—some regions densely planted, others relatively sparse—a distribution essential for evaluating the density-aware cost modulation. The UAV platform simulates a quadrotor (m=0.98kg, arm length 0.26m) equipped with a forward-facing depth camera (640×480, pinhole model, $90^{\circ }\times 73.7^{\circ }$ FOV, maximum sensing range 5.0m). The planner enforces $v_{max}=2.0\;m/s$ and $a_{max}=3.0\;{m/s}^{2}$; the initial flight altitude is set to 0.5m (below the canopy). The voxel map resolution is δ=0.1m. The environment layout is shown in Figure 4.

**Figure 5:**
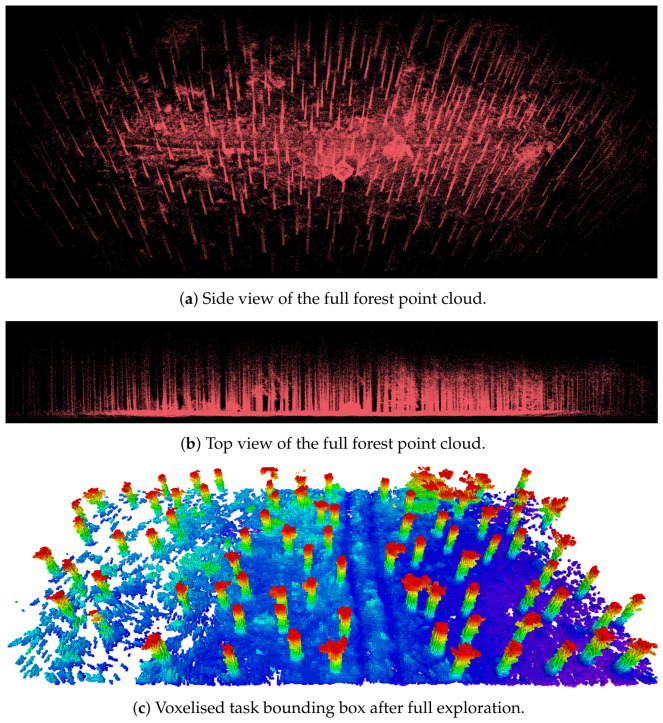
Point-cloud environment II reconstructed from a real forest scene [5]. (**a**,**b**) The full point-cloud map (64.5m×140.7m×17.9m) viewed from the side and top, respectively. (**c**) The voxelised occupancy map of the task bounding box (40m×19m×2m, below canopy) after complete exploration, containing N=70 tree trunks with non-uniform spacing and higher overall density than Environment I; occupied voxels are colour-coded by height.

To evaluate the generalisability of ORACLE across different tree densities, we introduce a second simulation environment (Environment II), reconstructed from a separate real-forest point cloud published by Jelavic et al. [5]. Environment II shares the identical task bounding box (40m×19m×2m) and UAV configuration as Environment I, but contains N=70 tree trunks at a markedly higher overall density ($\bar{n}=2.19$ vs. 1.56 trunks per planning cell). To quantify the spatial distribution difference, we uniformly divide the task area into 4×8=32 planning cells (5m×5m each). Let $n_{i}$ be the trunk count in cell *i* and K=32 the total number of cells. The mean density is $\bar{n}=N/K$, and three complementary metrics are defined as:

(11) $$\sigma =\sqrt{\frac{1}{K}\sum_{i=1}^{K}{\left(n_{i}-\bar{n}\right)}^{2}},\;CV=\frac{\sigma }{\bar{n}},\;VMR=\frac{\sigma^{2}}{\bar{n}}$$

where σ is the standard deviation, CV (coefficient of variation) measures relative dispersion and enables direct comparison across environments with different mean densities, and VMR (variance-to-mean ratio) characterises the spatial aggregation pattern (VMR=1: Poisson random; VMR>1: clustered; VMR<1: tending toward regular). Environment II has a higher VMR (0.70 vs. 0.56) and comparable CV (0.57 vs. 0.60), indicating a more aggregated trunk distribution with similar relative dispersion. This density-and-distribution contrast provides a complementary test condition for the density-aware cost modulation. The environment layout is shown in Figure 5.

**Figure 6:**
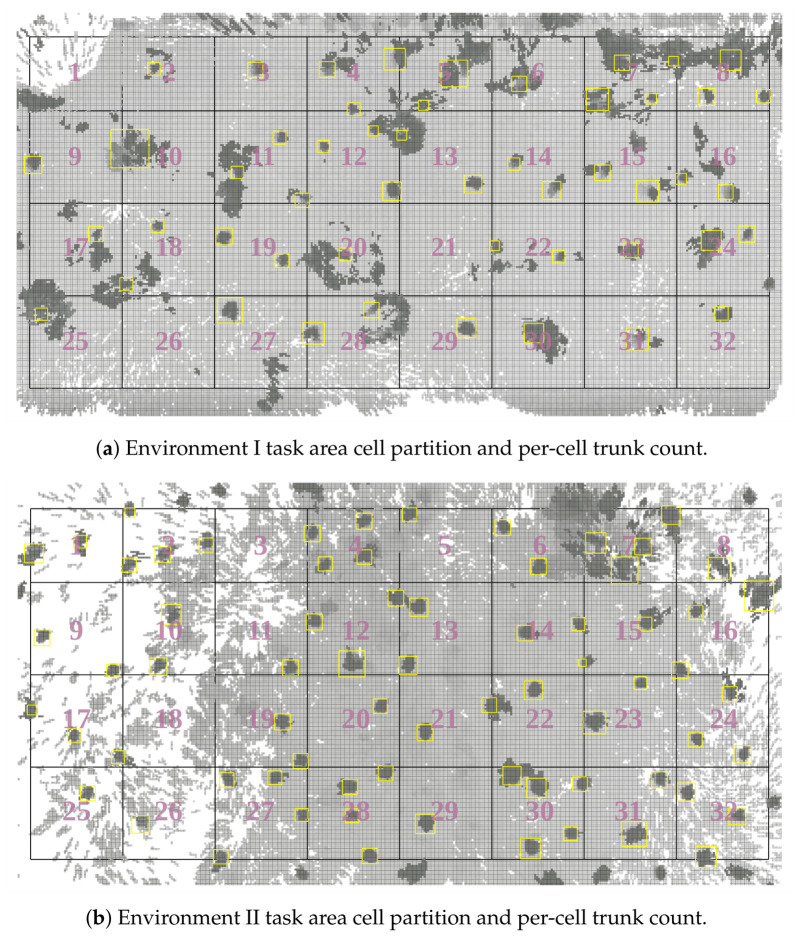
Top-down cell-partition maps of the two simulation environments. The 40m×19m task area is uniformly divided into 4×8=32 planning cells (numbered 1–32, row-major); each cell’s yellow detection box marks the tree trunks it contains. (**a**) Environment I (N=50, $\bar{n}=1.56$, CV=0.60, VMR=0.56). (**b**) Environment II (N=70, $\bar{n}=2.19$, CV=0.57, VMR=0.70).

Figure 6 visualises the per-cell trunk count in both environments, and Table 1 lists the numerical values. Environment I has $\bar{n}=1.56$ trunks per cell, σ=0.93, CV=0.60, and VMR=0.56; Environment II is markedly denser with $\bar{n}=2.19$, σ=1.24, CV=0.57, and VMR=0.70, the higher VMR reflecting a more aggregated spatial distribution. This density-and-distribution contrast provides complementary test conditions for evaluating the density-aware cost modulation.

Exploration terminates when two conditions hold simultaneously: (1) no frontiers remain (all reachable space has been observed), and (2) no UNVISITED targets exist in the global list. Condition (2) prevents premature termination when frontiers are exhausted—as long as unvisited trunks remain, the planning loop continues (cf. zone reactivation in Section 5.1.1).

Upon mission completion, several evaluation metrics are recorded. The final target coverage $C(T_{total})$ is the primary completeness indicator, evaluated at the mission termination time $T_{total}$. The target coverage rate is defined as the ratio of visited trunks to the ground-truth total:

(12) $$C\left(t\right)=\frac{N_{visited}\left(t\right)}{N_{gt}}\times 100\%$$

where $N_{gt}=50$ is the ground-truth trunk count and $N_{visited}\left(t\right)$ is the number of trunks marked VISITED by time *t*.

Online planning inevitably produces suboptimal paths—the UAV makes routing decisions with only partial target information, and each replanning cycle operates on a local snapshot rather than the global picture. $L_{flight}$ denotes the total flight distance accumulated from trajectory sample points. $L_{obs}$ is the sequential observation cost—the Euclidean chain $p_{start}\to obs_{1}\to obs_{2}\to \ldots$ in the actual visit order. As a post hoc benchmark, $L_{obs}^{*}$ is the LKH-optimal TSP tour [40] that visits the depot plus every actually-visited observation point; it represents the shortest possible tour under full global knowledge and therefore serves as a lower bound.

Two derived ratios distil these raw distances into interpretable efficiency indicators:

- Mission overhead ratio $\eta_{fo}=L_{flight}\;/\;L_{obs}^{*}\times 100\%$. Because $L_{flight}$ includes all flight segments—exploration sweeps, dead-leg returns, and productive observation legs—while $L_{obs}^{*}$ retains only the optimal observation tour, the ratio $\eta_{fo}$ captures the total overhead an online planner pays for the lack of global target information. A value of $\eta_{fo}=130\%$ means the UAV flies 30% more than the theoretically shortest observation tour; the closer $\eta_{fo}$ approaches 100%, the more the planner’s online decisions resemble the globally optimal route.
- Flight utilisation ratio $\eta_{util}=L_{obs}$/$L_{flight}\times 100\%$. This ratio measures the fraction of the actual flight distance that contributes directly to target observation. A high $\eta_{util}$ indicates the UAV spends most of its flight budget on productive observation legs rather than on exploration-related detours; a low value signals excessive non-observation flight.

**Figure 7:**
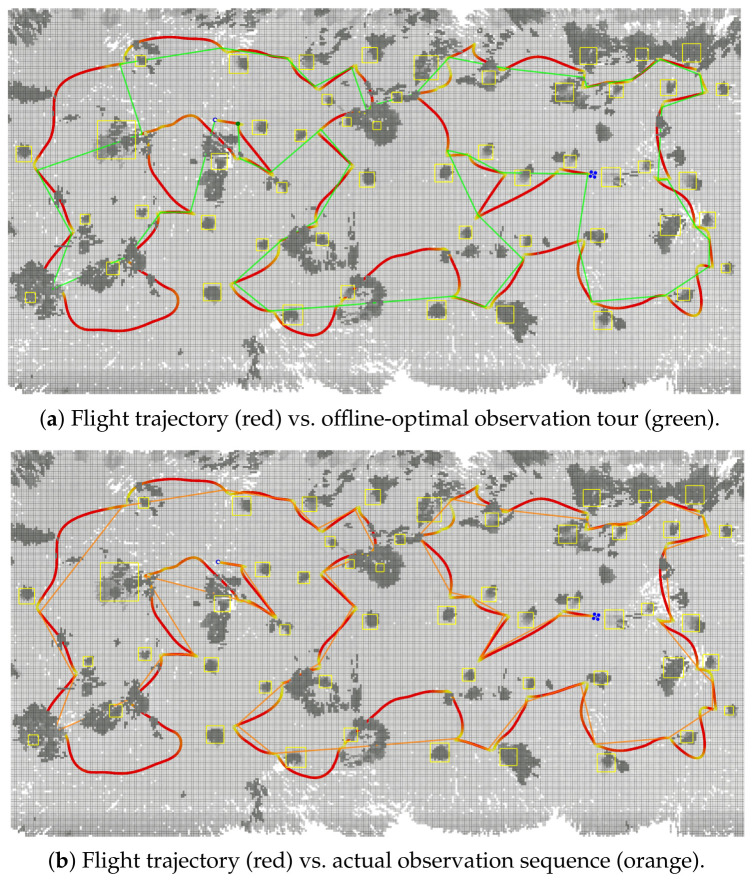
Top-down path comparison for a representative ORACLE run. The blue sphere marks the shared starting position (depot); yellow boxes denote visited tree trunks. (**a**) $L_{flight}$ (red) superimposed with the LKH-optimal tour $L_{obs}^{*}$ (green); the dark green sphere marks the optimal tour endpoint. (**b**) $L_{flight}$ (red) superimposed with the actual observation-point chain $L_{obs}$ (orange), connected in chronological visit order; the UAV icon marks the shared endpoint.

Taken together, $\eta_{fo}$ and $\eta_{util}$ provide complementary perspectives: $\eta_{fo}$ benchmarks the total mission cost against the offline optimum, while $\eta_{util}$ reveals the internal composition of each flight by separating observation work from exploration overhead. Figure 7 provides a geometric illustration of these two ratios for a representative ORACLE run. In Figure 7a, the red curve is the full executed flight trajectory $L_{flight}$ and the green polyline is the offline-optimal observation tour $L_{obs}^{*}$ computed post hoc by the LKH solver; the gap between the two paths reflects the mission overhead $\eta_{fo}$. In Figure 7b, the same red flight trajectory is overlaid with the orange polyline connecting observation points in the actual visit order ($L_{obs}$); segments where the red curve deviates from the orange chain represent non-observation flight, and the ratio between the orange and red path lengths corresponds to $\eta_{util}$.

### 6.2. Coverage Performance Comparison

The central question is whether switching the planning signal from “clearing unknown space” to “visiting discrete targets” yields substantive improvements in both coverage rate and path efficiency.

The primary comparison group is the FALCON+ baseline—the original FALCON [15] frontier-driven exploration augmented only with the tree-trunk detection module and visit-status bookkeeping (so that C(t) can be computed), but with none of ORACLE’s planning-guidance modules enabled: non-active zones containing unvisited trees are not reactivated into the global CP, no density weighting is applied to the ATSP cost matrix, and tree observation points are not injected into the SOP. In other words, FALCON+’s planning strategy is identical to vanilla FALCON; the detection layer merely provides a fair coverage-rate comparison. Both configurations run under identical environments and initial conditions. Each configuration is run n=5 times independently; mean and standard deviation are reported.

**Figure 8:**
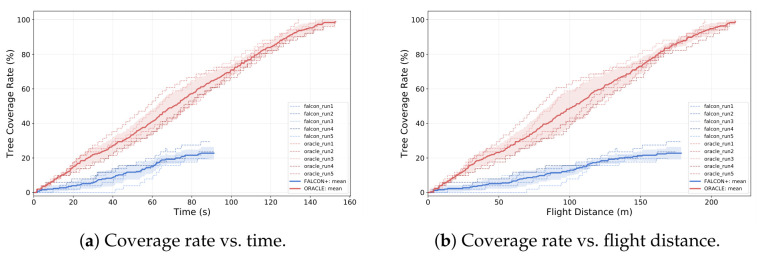
Target coverage rate comparison in Environment I (N=50) between FALCON+ baseline (blue) and ORACLE (red), n=5 runs each. Dashed lines show individual runs; solid lines indicate the mean; shaded regions denote ±1 standard deviation. (**a**) Coverage rate vs. time. (**b**) Coverage rate vs. flight distance.

**Figure 9:**
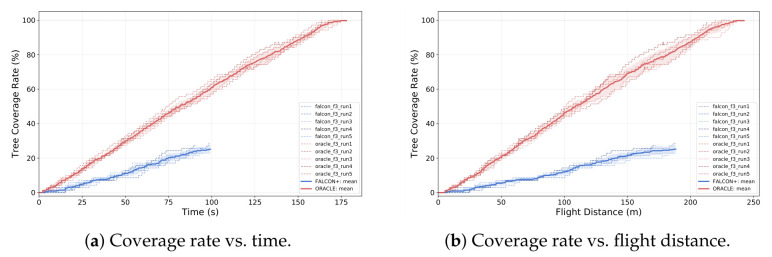
Target coverage rate comparison in Environment II (N=70) between FALCON+ baseline (blue) and ORACLE (red), n=5 runs each. Dashed lines show individual runs; solid lines indicate the mean; shaded regions denote ±1 standard deviation. (**a**) Coverage rate vs. time. (**b**) Coverage rate vs. flight distance.

If ORACLE’s design is correct, we expect two observable phenomena: (1) the FALCON+ baseline plateaus at some coverage level once frontiers are exhausted (with no incentive to re-visit zones containing unvisited targets), whereas ORACLE’s zone reactivation and target guidance continue to push C(t) toward 100%; (2) at any given coverage level, ORACLE’s cumulative flight distance is shorter, as density weighting reduces unnecessary detours and non-productive flight segments. Figure 8 and Figure 9 presents the coverage curves for both configurations, and the full numerical results are summarised in Table 2.

The results confirm all three predicted phenomena. ORACLE reaches a final coverage of 98.8±1.6%, whereas FALCON+ plateaus at only 22.7±3.6%—a gap directly caused by the absence of any target-seeking mechanism in the frontier-only baseline. The mission overhead ratio drops from 202.9% to 129.2%, meaning ORACLE’s total trajectory is only 29% longer than the offline-optimal observation tour, compared with a 103% excess for the baseline. Meanwhile, flight utilisation rises from 54.5% to 82.2%, confirming that a substantially larger share of ORACLE’s flight budget goes toward productive observation rather than exploration detours.

The above conclusions reproduce consistently in the denser Environment II (N=70). As shown in Figure 9, ORACLE achieves 99.7±0.6% terminal coverage vs. only 25.1±2.7% for FALCON+, with the coverage curve rising steadily toward near-100% while the baseline plateaus early. The mission overhead ratio $\eta_{fo}$ drops from 176.8% to 126.6%, and flight utilisation $\eta_{util}$ rises from 65.7% to 85.1%, with absolute improvements ($\Delta \eta_{fo}=50.2$ pp, $\Delta \eta_{util}=19.4$ pp) comparable to those in Environment I. The larger $L_{flight}$ and $L_{obs}$ in Environment II (225.2 m vs. 205.2 m; 191.6 m vs. 168.5 m) directly reflect the higher trunk count (N=70 vs. 50) and the correspondingly larger number of observation points. The consistent results across two environments with markedly different tree densities and spatial distributions confirm that the target-guided paradigm’s advantages are not sensitive to specific trunk count or density characteristics.

### 6.3. Ablation Study

To quantify the independent contribution of each planning-guidance module, we design two ablation variants (A1, A2), each disabling exactly one module while keeping the rest identical to the full ORACLE configuration. Each variant is run n=10 times, and we report mean ± std.

A1: w/o Zone Reactivation. This variant disables the zone reactivation mechanism (Section 5.1.1). With this module disabled, once a zone loses all its frontiers, any remaining unvisited targets within it become unreachable by the global planner. The primary comparison metric is final coverage $C(T_{total})$, which directly reflects the contribution of zone reactivation to coverage completeness. We expect the coverage curve to exhibit a pronounced “terminal plateau”—$C(T_{total})$ drops noticeably below the full ORACLE, with several trees permanently excluded from the global route. Because disabling this module causes a substantial fraction of targets to remain permanently unvisited, the resulting flight cost data reflects an incomplete mission and is not directly comparable to the full-coverage runs; cost-related metrics are therefore omitted for A1.

A2: w/o Density Weighting. This variant degenerates all zone cost weights to $w_{j}\equiv 1$ (i.e., $\alpha =0,\;\beta_{0}=0$ in Equation (5)). The ATSP and SOP solvers then rank zones purely by flight cost. Without density-aware weighting, the planner cannot distinguish high-density from sparse regions, causing the UAV to make more non-productive out-and-back traversals by entering zones with few targets and then returning. The primary evaluation metrics for A2 are the efficiency ratios $\eta_{fo}$ and $\eta_{util}$, together with coverage convergence speed, quantifying the impact of density weighting on how effectively the flight budget is allocated. We expect final coverage to remain comparable to the full ORACLE (targets are still visited sequentially), but the mission overhead ratio $\eta_{fo}$ worsens and the flight utilisation ratio $\eta_{util}$ drops. If enabling density weighting lowers $\eta_{fo}$ (closer to 100%) and raises $\eta_{util}$, this confirms that density information effectively steers the limited flight budget toward high-value regions, reducing exploration overhead and improving flight resource utilisation.

**Figure 10:**
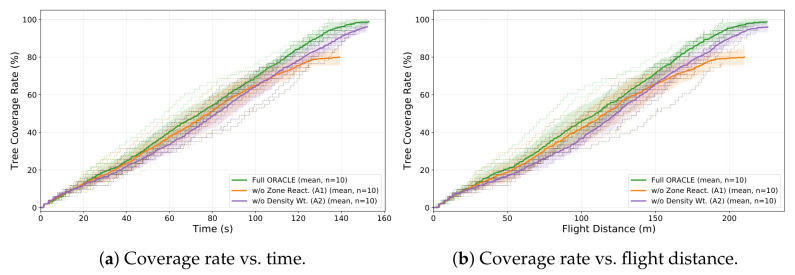
Target coverage-rate comparison in the ablation study (Environment I, N=50). Full ORACLE is shown in green, A1 w/o zone reactivation in orange, and A2 w/o density weighting in purple, with n=10 independent runs for each configuration. Dashed lines show individual runs; solid lines indicate the mean; shaded regions denote ±1 standard deviation. (**a**) Coverage rate vs. time. (**b**) Coverage rate vs. flight distance.

**Figure 11:**
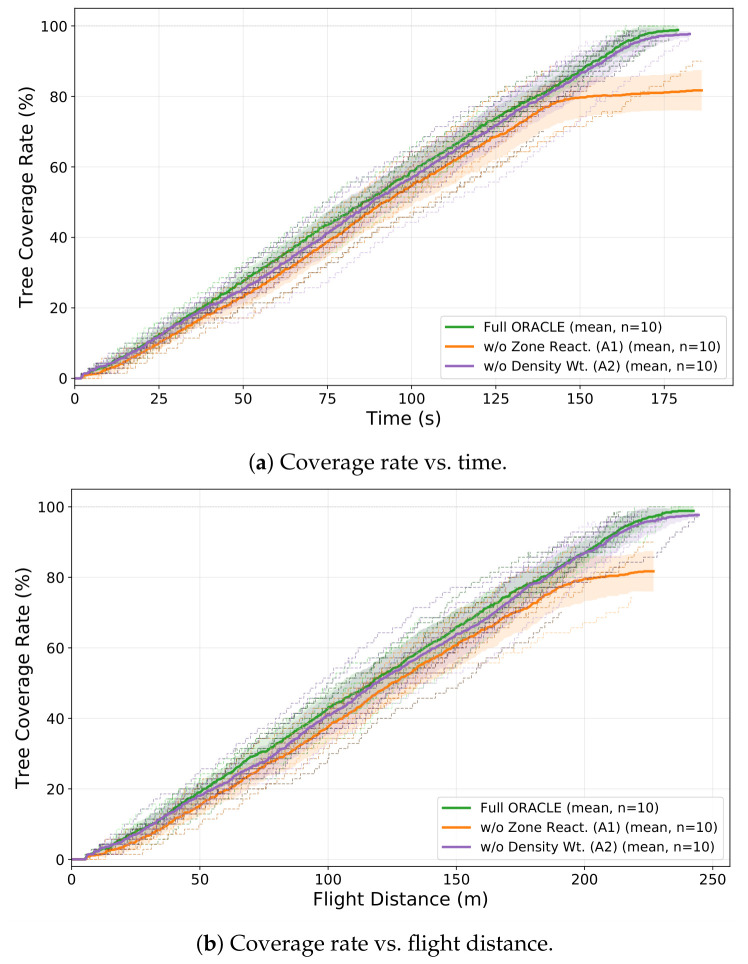
Target coverage-rate comparison in the ablation study (Environment II, N=70). Full ORACLE (green), A1 w/o zone reactivation (orange), A2 w/o density weighting (purple), n=10 runs each. Dashed lines: individual runs; solid lines: mean; shaded bands: ±1 standard deviation. (**a**) Coverage rate vs. time. (**b**) Coverage rate vs. flight distance.

Figure 10 and Figure 11 shows the coverage curves of the three configurations, and the corresponding numerical results are summarised in Table 3.

Figure 11 presents the ablation results in Environment II (N=70). The findings are generally consistent with Environment I.

As Figure 10 shows, the A1 curve separates from the full ORACLE around t=100 s and saturates near 80%. Disabling zone reactivation drops the final coverage from 98.8% to 80.0%—a reduction of 18.8 percentage points—confirming that zone reactivation is the decisive factor for coverage completeness. Without it, roughly 10 out of 50 trunks are permanently stranded in non-active zones and never re-entered by the global planner.

The A2 curve in Figure 10 follows the full ORACLE trend closely, but under the same flight budget (time or flight distance), it consistently attains a lower coverage level. Disabling density weighting reduces coverage by 2.7 percentage points (98.8%→96.1%) and worsens both efficiency ratios: $\eta_{fo}$ rises from 129.3% to 133.7% (+4.4 pp), meaning the UAV pays 4.4% more overhead relative to the offline optimum, while $\eta_{util}$ drops from 81.9% to 79.7% (−2.2 pp), indicating a smaller fraction of the flight budget goes toward productive observation.

In Environment II (Figure 11, Table 3), the ablation conclusions are generally consistent. A1 (w/o zone reactivation) separates from the full ORACLE curve around t=110 s and saturates near 82%, with final coverage dropping from 98.9% to 81.7% (−17.2 pp), closely matching the −18.8 pp reduction observed in Environment I. This confirms that zone reactivation is the decisive factor for coverage completeness, regardless of tree density. The larger standard deviation of A1 in Environment II (5.7% vs. 4.4%) reflects greater run-to-run variability in which specific trunks become stranded in the denser environment. A2 (w/o density weighting) follows the full ORACLE trend but with a consistent lag: final coverage drops by 1.2 pp (98.9%→97.7%). In this significantly denser environment, the efficiency ratio differences are attenuated—$\eta_{fo}$ is 127.0% vs. 126.0%, and $\eta_{util}$ is 84.9% vs. 86.1%—indicating a marginal diminishing-return effect for density weighting when the overall trunk density is high and the distribution contrast is less pronounced (VMR=0.70 vs. 0.56). This is consistent with the expectation that density weighting yields larger benefits in environments with stronger density contrast.

A notable feature of Table 3 is that the three absolute distance metrics—$L_{flight}$, $L_{obs}$, and $L_{obs}^{*}$—are close between the full ORACLE and A2 (e.g., $L_{flight}$: 205.2 m vs. 206.8 m). This does not imply that density weighting has little effect. The scene is a bounded 40m×19m box; both variants must traverse it nearly in full before termination, so total flight distance is largely dictated by the environment footprint, not by which zones are visited first. What density weighting changes is the allocation of that distance budget: it steers the UAV toward high-density zones earlier, converting more flight into productive observation and reducing non-productive traversals through sparse regions. Moreover, because the full ORACLE visits more targets (∼50 trunks vs. ∼49), both $L_{obs}$ and $L_{obs}^{*}$ are actually higher for the full ORACLE, yet the ratio $\eta_{fo}=L_{flight}/L_{obs}^{*}$ remains lower because the denominator grows faster than the small increase in the numerator. In short, the full ORACLE accomplishes a harder task—visiting more targets to a higher coverage standard—within essentially the same flight budget. The ratio metrics therefore normalise for the coverage-level difference and correctly isolate the per-unit-work efficiency gain that the raw distances cannot reveal.

Moreover, the moderate magnitude of the A2 improvement is related to two factors. First, the current density-weight parameters (α=1.4, $\beta_{0}=0.65$ in Equation (5)) adopt a relatively balanced setting that avoids aggressive down-weighting of sparse zones; pushing toward a more aggressive boundary (larger α or $\beta_{0}$) could amplify the effect, but it would also further weaken the role of baseline flight cost in global guidance and may cause the planner to neglect nearby targets in favour of farther dense regions. Second, the degree of non-uniformity in the target distribution of the current test environment limits the margin available for density-driven re-routing—in a scene whose density contrast is more pronounced, the benefit is expected to be larger. A systematic study of parameter sensitivity and environment-dependent performance is left for future work.

Beyond the final-coverage gap, the coverage convergence speed also differs, and A2 exhibits a clear lag at all coverage levels. At a fixed flight distance of 100 m (roughly half the mission budget), the full ORACLE has already covered 45.7% of all targets on average, whereas A2 reaches only 36.7%—a deficit of 9.0 pp. Equivalently, to reach the 90% coverage milestone, A2 requires 198.1±7.0 m of flight vs. ORACLE’s 184.8±7.1 m, representing 13.3 m (7.2%) more distance, and 136.1±5.9 s vs. 127.2±4.3 s, representing 8.9 s (7.0%) more time. This persistent mid-mission lag, visible as the purple curve remaining below the green curve throughout Figure 10, confirms that density weighting accelerates target acquisition by prioritising high-density regions. The time and distance required to reach each coverage milestone are summarised in Table 4; A1 never reaches 90% because some targets remain permanently stranded. In Environment II, the convergence-speed advantage of density weighting is attenuated: to reach 90% coverage, A2 requires 154.4±8.7 s vs. ORACLE’s 153.4±4.7 s (+1.0 s, <1%), and 206.0±12.6 m vs. 204.2±8.3 m (+1.8 m). This narrowing gap is consistent with the higher baseline density in Environment II ($\bar{n}=2.19$ vs. 1.56): when targets are abundant everywhere, the marginal benefit of prioritising high-density zones over sparse ones naturally diminishes. Conversely, in Environment I where the density contrast is more operationally meaningful (VMR=0.56 indicating a more regular-like distribution with distinct sparse clusters), the re-routing effect is more pronounced. A1 fails to reach the 90% threshold in most runs for both environments, further confirming that zone reactivation is indispensable for coverage completeness independent of tree density.

## 7. Conclusions

We presented ORACLE, an object-centric autonomous coverage exploration framework that shifts the planning paradigm from space-guided to target-guided exploration for efficient discrete-obstacle inspection in unknown environments. The design rests on three cooperating modules: an online detection and persistent identification module based on occupied-voxel CCL provides real-time target perception; a density-aware ATSP global planner steers the UAV toward high-value regions at the zone level; and a target-guided SOP local planner produces observation sequences that replace the traditional space-voxel-clearing objective with discrete-object traversal, and en-route VP inclusion maintains the balance between spatial exploration and target traversal. The three layers of target awareness form a complete perception-to-decision loop, enabling the system to explore and traverse simultaneously in fully unknown environments.

Experimental results demonstrate that: (1) The target-guided paradigm substantially improves both target coverage rate and path efficiency across two environments with contrasting tree densities—ORACLE achieves 98.8% (Environment I) and 99.7% (Environment II) final coverage vs. 22.7% and 25.1% for the space-guided FALCON+ baseline, with the coverage curve rising steadily toward near-100% while the baseline plateaus once frontiers are exhausted. The mission overhead ratio $\eta_{fo}$ drops from 202.9% to 129.2% (Environment I) and from 176.8% to 126.6% (Environment II), confirming that replacing frontier viewpoints with trunk observation points in the SOP fundamentally aligns the trajectory with inspection objectives. (2) Ablation studies confirm independent contributions of each module across both environments—disabling zone reactivation drops coverage by 18.8 percentage points (Environment I, to 80.0%) and 17.2 percentage points (Environment II, to 81.7%), while disabling density weighting worsens $\eta_{fo}$ by 4.4 percentage points (Environment I). Furthermore, at the same flight distance of 100 m in Environment I, the full ORACLE already covers 9.0 percentage points more targets than the variant without density weighting (45.7% vs. 36.7%), confirming that density-aware cost modulation accelerates target acquisition by prioritising high-density regions.

Current limitations and future directions: (1) The target detector relies on the geometric prior of approximately cylindrical trunks and is therefore best-suited to managed orchards and plantation forests where trunk regularity is a reasonable operational assumption; for irregularly shaped targets (e.g., fallen trees, leaning trunks, forked stems) or dense understory vegetation, the current CCL-plus-filter pipeline may produce missed or false detections. A promising direction is to integrate lightweight learning-based detectors (e.g., point-cloud segmentation networks) to broaden applicability to unmanaged natural forests while maintaining online computational efficiency. (2) The current traversal trajectory remains segmented, with deceleration or brief pauses at observation points, limiting mission time efficiency. Future work will investigate smooth continuous-curve trajectories that pass through the shared valid-observation zones of multiple targets at non-zero speed, layered on top of the current framework without altering the core planning paradigm, achieving a better trade-off between coverage quality and flight fluency. (3) The present study validates ORACLE in a simulation environment reconstructed from a real forest point cloud (MARSIM) [42], which isolates algorithmic performance and enables statistically rigorous multi-run comparisons. Real-world field validation under forest canopies is planned as the immediate next step, for which a complete hardware–software deployment pipeline has been drafted. The aerial platform is a custom-built quadrotor (Totem Q250 frame, 250 mm wheelbase) equipped with an Intel NUC12 onboard computer (Core i5-1240P, 16 GB RAM), a Pixhawk4 flight controller running PX4 firmware, and an Intel RealSense D435 stereo depth camera for onboard perception. The software stack integrates VINS-Fusion for visual–inertial state estimation and MAVROS as the communication bridge between the onboard computer and the flight controller. (4) A rigorous study of computational scalability across diverse scene scales and tree densities is planned to establish the practical operational envelope of the online ATSP replanning loop. Moreover, a systematic parameter sensitivity analysis across diverse environment configurations and the development of automated, possibly online-adaptive, parameter-tuning methodology for the density-aware cost modulation are also identified as valuable future investigations. (5) ORACLE currently operates in a single-UAV setting; extending the target-guided paradigm to multi-UAV cooperative coverage (including task allocation and conflict resolution) represents another direction of interest.

## Figures and Tables

**Table 1:** Per-cell trunk count in the two simulation environments.

Cell ID	1	2	3	4	5	6	7	8	9	10	11	12	13	14	15	16
Env. I	0	1	1	3	2	1	4	3	1	1	3	3	2	2	2	2
Env. II	2	4	0	4	1	2	4	1	2	2	1	3	2	2	2	3
Cell ID	17	18	19	20	21	22	23	24	25	26	27	28	29	30	31	32
Env. I	1	2	2	1	0	2	1	2	1	0	1	2	1	1	1	1
Env. II	3	0	2	1	2	1	2	3	1	1	4	4	1	5	2	3

**Table 2:** Coverage performance comparison (n=5 runs each; mean ± std. dev.).

Method	$C(T_{\mathrm{total}})$ (%)	$L_{\mathrm{flight}}$ (m)	$L_{\mathrm{obs}}$ (m)	$L_{\mathrm{obs}}^{*}$ (m)	$\eta_{\mathrm{fo}}$ (%)	$\eta_{\mathrm{util}}$ (%)	$T_{\mathrm{total}}$ (s)
Environment I (N=50)							
FALCON+ Baseline	22.7±3.6	165.7±5.8	90.7±16.8	82.6±10.7	202.9±18.5	54.5±8.3	91.9±1.9
ORACLE (ours)	98.8±1.6	205.2±6.7	168.5±4.3	159.1±4.0	129.2±7.3	82.2±2.8	147.9±6.7
Environment II (N=70)							
FALCON+ Baseline	** 25.1±2.7 **	** 174.4±7.5 **	** 114.6±6.1 **	** 98.9±4.1 **	** 176.8±12.6 **	** 65.7±2.4 **	** 103.8±2.5 **
ORACLE (ours)	** 99.7±0.6 **	225.2±11.4	191.6±8.8	177.9±3.3	126.6±5.3	85.1±1.2	176.9±5.8

**Table 3:** Ablation study results (n=10 runs each; mean ± std. dev.).

Variant	$C(T_{\mathrm{total}})$ (%)	$L_{\mathrm{flight}}$ (m)	$L_{\mathrm{obs}}$ (m)	$L_{\mathrm{obs}}^{*}$ (m)	$\eta_{\mathrm{fo}}$ (%)	$\eta_{\mathrm{util}}$ (%)
Environment I (N=50)						
Full ORACLE	98.8±1.3	205.2±9.2	168.0±5.5	158.7±3.6	129.3±6.3	81.9±2.2
w/o Zone React. (A1)	80.0±4.4	—	—	—	—	—
w/o Density Wt. (A2)	96.1±2.9	206.8±9.2	164.8±6.6	154.7±5.0	133.7±5.5	79.7±2.1
Environment II (N=70)						
Full ORACLE	98.9±1.3	222.5±9.8	188.8±7.9	175.3±3.9	126.0±5.7	86.1±1.0
w/o Zone React. (A1)	81.7±5.7	—	—	—	—	—
w/o Density Wt. (A2)	97.7±0.7	219.1±10.0	188.7±7.9	174.0±2.9	127.0±5.2	84.9±2.8

**Table 4:** Convergence speed comparison: Time (s) and flight distance (m) to reach each coverage threshold (n=10 runs; mean ± std. dev.). A dash (—) indicates the threshold was never reached.

Variant	Time to Threshold (s)				Distance to Threshold (m)			
	50%	70%	80%	90%	50%	70%	80%	90%
Environment I (N=50)								
Full ORACLE	74.2±7.7	98.9±6.1	113.5±4.0	127.2±4.3	109.7±15.2	144.2±11.5	164.1±7.4	184.8±7.1
w/o Zone React. (A1)	78.1±9.1	107.3±7.8	121.3±5.3 (6/10)	—	116.6±16.1	159.1±14.8	178.5±10.5 (6/10)	—
w/o Density Wt. (A2)	82.4±7.6	105.8±7.2	121.4±6.6	136.1±5.9	124.6±9.3	155.4±8.3	177.0±7.9	198.1±7.0
Environment II (N=70)								
Full ORACLE	84.7±6.2	117.1±4.7	135.4±6.5	153.4±4.7	114.7±11.2	157.2±8.7	181.1±10.3	204.2±8.3
w/o Zone React. (A1)	91.9±8.7	125.8±11.8	140.5±11.3	—	124.9±12.3	170.1±17.8	188.5±9.4	—
w/o Density Wt. (A2)	87.5±9.5	120.8±10.2	137.1±9.0	154.4±8.7	117.7±12.5	161.8±14.4	182.7±12.4	206.0±12.6

## Data Availability

The original contributions presented in this study are included in the article. Further inquiries can be directed to the corresponding author.

## Abbreviations

The following abbreviations are used in this manuscript:

ORACLE	Object-centric Autonomous Coverage Exploration
UAV	Unmanned Aerial Vehicle
ATSP	Asymmetric Travelling Salesman Problem
SOP	Sequential Ordering Problem
CCL	Connected Component Labeling
CPP	Coverage Path Planning
TSP	Travelling Salesman Problem
CP	Coverage Path
VP	Viewpoint
DBH	Diameter at Breast Height
TLS	Terrestrial Laser Scanning
ULS	Under-canopy UAV Laser Scanning
MMS	Mobile Mapping System
PLS	Personal Laser Scanning
LKH	Lin–Kernighan–Helsgaun Heuristic
ROS	Robot Operating System
FOV	Field of View
SLAM	Simultaneous Localisation and Mapping
